# Green synthesis, characterization, and antimicrobial applications of silver nanoparticles as fluorescent nanoprobes for the spectrofluorimetric determination of ornidazole and miconazole

**DOI:** 10.1038/s41598-022-25830-x

**Published:** 2022-12-10

**Authors:** Galal Magdy, Eman Aboelkassim, Ramadan A. El-Domany, Fathalla Belal

**Affiliations:** 1 Pharmaceutical Analytical Chemistry Department, Faculty of Pharmacy, Kafrelsheikh University, P.O. Box 33511, Kafrelsheikh, Egypt; 2 Microbiology and Immunology Department, Faculty of Pharmacy, Kafrelsheikh University, P.O. Box 33511, Kafrelsheikh, Egypt; 3 Pharmaceutical Analytical Chemistry Department, Faculty of Pharmacy, Mansoura University, P.O. Box 35516, Mansoura, Egypt

**Keywords:** Analytical chemistry, Fluorescent probes

## Abstract

A green and simple method was proposed for the synthesis of silver nanoparticles (Ag-NPs) using *Piper cubeba* seed extract as a reducing agent for the first time. The prepared Ag-NPs were characterized using different spectroscopic and microscopic techniques. The obtained Ag-NPs showed an emission band at 320 nm when excited at 280 nm and exhibited strong green fluorescence under UV-light. The produced Ag-NPs were used as fluorescent nanosensors for the spectrofluorimetric determination of ornidazole (ONZ) and miconazole nitrate (MIZ) based on their quantitative quenching of Ag-NPs native fluorescence. The current study introduces the first spectrofluorimetric method for the determination of the studied drugs using Ag-NPs without the need for any pre-derivatization steps. Since the studied drugs don't exhibit native fluorescent properties, the importance of the proposed study is magnified. The proposed method displayed a linear relationship between the fluorescence quenching and the concentrations of the studied drugs over the range of 5.0–80.0 µM and 20.0–100.0 µM with limits of detection (LOD) of 0.35 µM and 1.43 µM for ONZ and MIZ, respectively. The proposed method was applied for the determination of ONZ and MIZ in different dosage forms and human plasma samples with high % recoveries and low % RSD values. The developed method was validated according to ICH guidelines. Moreover, the synthesized Ag-NPs demonstrated significant antimicrobial activities against three different bacterial strains and one candida species. Therefore, the proposed method may hold potential applications in the antimicrobial therapy and related mechanism research.

## Introduction

*Piper cubeba* is a plant cultivated in Java and Sumatra for its essential oil. It was used for the treatment of abdominal pain and dysentery. *Piper cubeba* has many active constituents including tannins, glycosides, and polyphenols as flavonoids that is responsible for its antioxidant and reducing activities^1^.

The synthesis of silver nanoparticles (Ag-NPs) have attracted the interest of many researchers because of their different applications, including environmental^2,3^, industrial^4,5^, biomedical^6,7^, and pharmaceutical applications^8,9^. Ag-NPs synthesis could be performed by two methods, either “top to down” or “bottom to top”. Top to down approach includes the starting material size reduction where nanoparticles usually made by several physical techniques^10,11^. Bottom to top method is performed using a reducing and/or capping agent that could be chemical or natural. The main drawback of using chemical agents was their potential toxicity that made researchers directed to the green synthesis of nanoparticles using either animal metabolites^12,13^, bacteria^14^, fungus^15^, algae^16^, or plants^17^.

Ag-NPs were found to have strong antimicrobial activity against various microorganisms including, fungi, virus, and bacteria^18,19^. Due to their antibacterial activities, Ag-NPs have recently been used extensively in a variety of disciplines including agriculture^20^, water disinfection^21^, textiles and paints^22,23^, and as an antimicrobial agent in combination with other antibiotics^24^.

**Figure 1:**
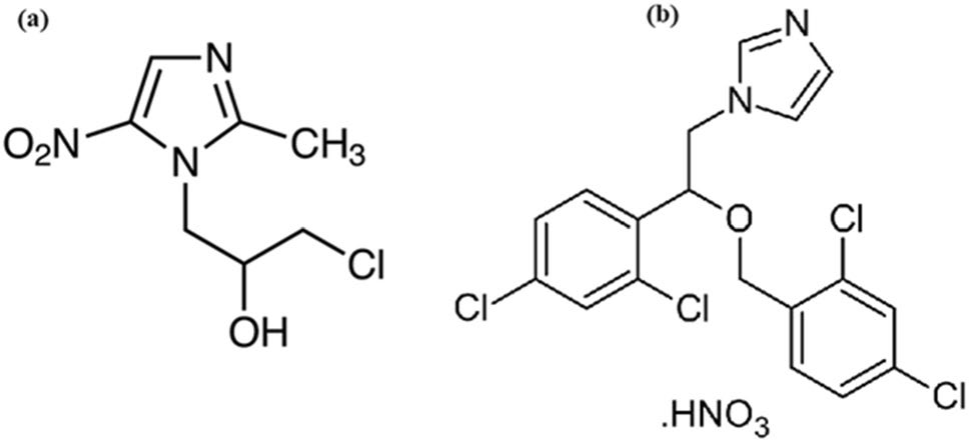
Chemical structure of ONZ (**a**) and MIZ (**b**).

Ornidazole (ONZ) is a nitroimidazole drug that has been used to treat bacterial and protozoal infections. ONZ has been also used for treating the infections of the intestine, stomach, and uro-genital tract. The chemical name of ONZ is 1-(3-chloro-2-hydroxypropyl)-2-methyl-5-nitroimidazole (Fig. 1a)^25^. Miconazole nitrate (MIZ) is an imidazole antifungal drug that has been used for the treatment of skin infections and superficial candidiasis. MIZ could also be taken orally or as oral gel for treating intestinal and oropharyngeal candidiasis. Chemically, it is 1‐[2,4‐Dichloro‐b‐(2,4‐dichlorobenzyloxy) phenylethyl]‐1H‐imidazole (Fig. 1b)^26^. Both ONZ and MIZ belong to anti-microbial drugs.

Many techniques have been reported in the literature for determining ONZ and MIZ in pharmaceutical dosage forms. The reported techniques for ONZ were spectrophotometry^27–29^, spectrofluorimetry^30^, HPLC^31,32^, TLC^33^, electrochemistry^34–37^, gas chromatography^38^, and capillary electrophoresis^39,40^, while for MIZ the reported methods included spectrophotometry^41,42^, gas chromatography^38,43^, capillary zone electrophoresis^44^, HPLC^45–48^, and HPTLC^49^. Till now, there haven’t been any reported spectrofluorimetric methods for the determination of either ONZ or MIZ using Ag-NPs and no spectrofluorimetric methods at all were reported for MIZ determination.

The current study aimed to discuss the ecofriendly synthesis of Ag-NPs using *Piper cubeba* seed extract as a green reducing agent for the first time. The characterization of Ag-NPs optical properties and morphology was carried out. Antimicrobial activities had also been investigated. In addition, the quenching of fluorescence intensity by ONZ and MIZ was examined and used to design a simple and green spectrofluorimetric method for their estimation. Since the studied drugs don't exhibit native fluorescent properties, the importance of the proposed study is magnified as it presents a simple, green, and sensitive spectrofluorimetric approach for their analysis in different dosage forms and human plasma samples. Compared to the proposed method, the reported spectrofluorimetric method for ONZ^30^ required complicated and time consuming steps. This method is considered the first spectrofluorimetric one for MIZ.

## Experimental

### Materials and reagents

Ornidazole (99.16%) was provided by Organopharma (Cairo, Egypt) and miconazole nitrate (99.95%) was kindly provided by Amriya Pharmaceutical industries (Alexandria, Egypt). Astranida^®^ coated tablets (500 mg ONZ/tablet, Batch No. 19052A), Daktarin^®^ cream (20 mg MIZ/1 g, Batch No. LBE0841), and Daktarin^®^ oral gel (20 mg miconazole/1 g, Batch No. LIB2G00) were purchased from local Pharmacy. *Piper cubeba* seeds were purchased from local market. Human plasma samples were provided by Mansoura University Hospitals (Mansoura, Egypt), and they were stored at − 80 °C until used after thawing. Silver nitrate, phosphoric acid, methanol, boric acid, and glacial acetic acid were purchased from Sigma-Aldrich (St. Louis, MO, USA). Britton–Robinson buffer (0.02 M, pH 2–12) solutions were freshly prepared. All reagents and chemicals were of analytical grade and the experiments were performed using double distilled water.

### Preparation of piper cubeba extract

**Figure 2:**
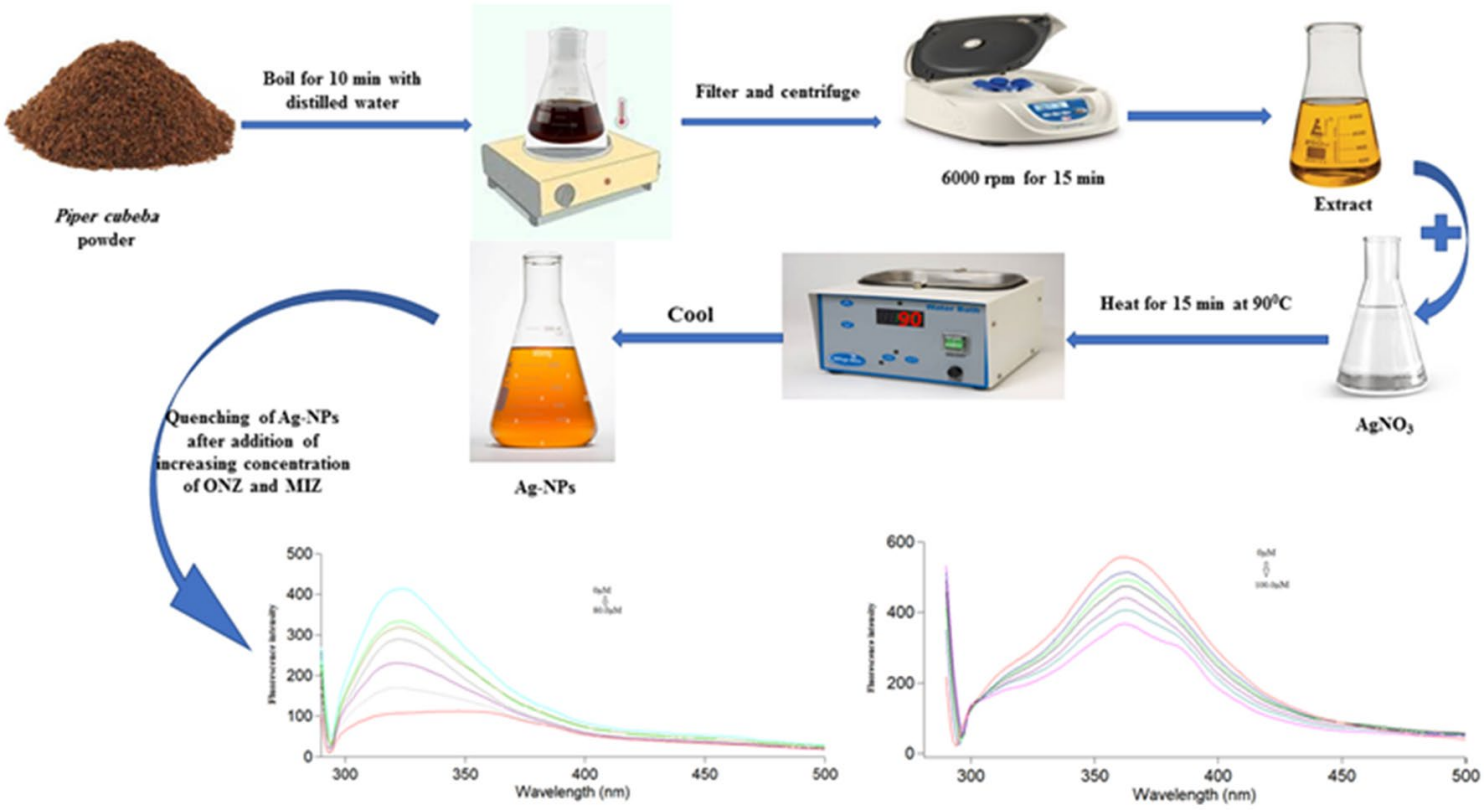
Scheme for the synthesis of Ag-NPs and application for determination of ONZ and MIZ.

*Piper cubeba* powder (5 g) was weighed and boiled for 10 min in 100 mL double distilled water. After cooling, the resultant solution was then filtered and centrifuged at 6000 rpm for 15 min then the supernatant was kept in refrigerator until used (Fig. 2). All experiments on *Piper cubeba* seeds were performed in accordance with relevant guidelines and regulations and were approved by the Committee of Research Ethics in the Faculty of Pharmacy, Kafrelsheikh University, Kafrelsheikh, Egypt.

### Synthesis of Ag-NPs

Ag-NPs were prepared using a green synthesis approach by mixing 10 mL (1.0 mM) AgNO_3_ with 5 mL *Piper cubeba* extract followed by heating for 15 min using water bath maintained at 90 °C. After cooling, the color turned dark brown indicating the formation of Ag-NPs. The prepared solution was stored in the refrigerator for later use (Fig. 2).

### Instrumentation

Fluorescence measurements were performed using Cary Eclipse Fluorescence Spectrophotometer with Xenon flash lamp from Agilent Technologies (United States). It was adjusted at 800 V and the slit width was set at 5 nm. A double beam spectrophotometer (PG Instrument, UK) was used to carry out the spectrophotometric measurements. The Fourier Transform Infrared (FT-IR) spectra of Ag-NPs were detected using FT-IR spectrophotometer (IS10 Nicolet, USA). All measurements were carried out in the range of 4000–1000 cm^−1^ and recorded at 4 cm^−1^ resolution as 32 scans. The morphology of Ag-NPs was investigated using high-resolution transmission electron microscope (HRTEM) operated at 200 kV (JEOL, JEM-2100, Tokyo). Size of Ag-NPs was also confirmed by using the dynamic light scattering (DLS) nanoparticle size analyzer (Brookhaven, USA). As well, elemental analysis of Ag-NPs was performed using energy-dispersive X-ray (EDX) spectroscopy attached to scanning electron microscope (Jeol, JSM-IT100LA, Japan). Centrifugation was performed using Centrifuge, model 2-16P, (Germany). For pH measurements, a Jenway 3510 pH meter (Jenway, UK) was used. Vortex mixer (IVM-300p, Taiwan) and ultrasonic bath (SS 101H 230, USA) were also used.

### Antimicrobial study

The antimicrobial activity of Ag-NPs synthesized from *Piper cubeba* extract was investigated using agar disc diffusion method against four different pathogenic organisms including, *Bacillus subtilis*, *Staphylococcus aureus, Escherichia coli,* and *Candida albicans*. The synthesized Ag-NPs (50.0 µg/mL) were tested for antimicrobial activity in comparison with the *Piper cubeba* extract and 1.0 mM of each of AgNO_3_, ONZ, and MIZ. The method used the Lysogenia Broth (LB) media where 100 µL of liquid culture were spread on, and then tested organisms were cultured on the LB agar plates. Filter paper discs containing the tested compounds were placed on the agar surface, and after incubation overnight at 27 °C, the germination and growth of the tested microorganisms were inhibited and the diameters of inhibition zones (mm) were measured and recorded.

### Standard solutions

Standard solution of each of ONZ and MIZ (1.0 mM) was prepared separately by dissolving 0.011 g and 0.024 g, respectively, in methanol in 50 mL volumetric flask. Serial dilution of the standard solution was performed using double distilled water to prepare working solutions in the range of 5.0–80.0 μM for ONZ and 20.0–100.0 μM for MIZ. The standard solutions of both drugs were stable for at least one week when kept at 4 °C.

### Spectrofluorimetric measurements

Factors that could affect the sensing of the studied drugs fluorescence were optimized by adding 0.5 mL of Ag-NPs to 20.0 μM of ONZ or 50.0 μM of MIZ solution. The intensity of fluorescence was determined at 320 nm using an excitation wavelength of 280 nm. The fluorescence intensities of increased concentrations of ONZ and MIZ in the range of 5.0–80.0 µM and 20.0–100.0 µM, respectively were investigated at room temperature, at pH of 4 and 9 using 2 mL and 1 mL Britton–Robinson buffer, respectively. The calibration curves were plotted using fluorescence quenching *vs.* the drugs concentrations (μM) then the regression equations were derived.

### Method optimization

Experimental parameters that could affect the quenching of the fluorescence intensity of Ag-NPs by ONZ and MIZ were studied. Such parameters included the buffer pH, buffer volume, incubation time, and temperature. The pH effect was studied using Britton–Robinson buffer over the pH range of 2–12. Buffer volume in the range of (250.0 µL–2.0 mL) for ONZ and (200.0 µL–1.5 mL) for MIZ was also investigated. The incubation time effect was studied at different time intervals from one to sixty minutes. Finally, the temperature was studied in the range of (25–60 °C) for ONZ and (25–40 °C) for MIZ.

### Method validation

The proposed method was validated according to ICHQ2 (R1) guidelines^50^. Validation parameters including linearity, limit of detection, limit of quantitation, accuracy, precision, robustness, and method selectivity were studied. Linearity was studied using six standard solutions (n = 3) over the range of (5.0–80.0 μM) and (20.0–100.0 μM) for ONZ and MIZ, respectively. The calibration curves were obtained by plotting fluorescence quenching (F_0 _− F) versus different drug concentrations in μM, and the data obtained was analyzed. Limits of detection (LOD) and quantitation (LOQ) are a measure of method sensitivity, and they could be calculated using the following Equations^51^:

1 $$LOD = 3.3\frac{{S_{a} }}{b}$$

2 $$LOQ = 10\frac{{S_{a} }}{b}$$

where S_a_ is the standard deviation of the intercept of the regression line and b is its slope.

The method accuracy was also investigated by comparing the results obtained by the proposed method to those obtained by the reported methods^29,42^. Method precision was also evaluated using three replicates of three different concentrations in the same day (intra-day precision) and during three consecutive days (inter-day precision). After that, the analytical data was statically analyzed using Student t-test and Variance ratio F test for accuracy and precision, respectively.

Robustness of the developed method was studied to find out if small changes in the experimental parameters could influence the quenching of the fluorescence intensities of Ag-NPs by the studied drugs. For ONZ, these changes involved: volume of Ag-NPs (500.0 μL ± 5), Britton–Robinson buffer pH (4 ± 0.2), and volume of buffer (2 mL ± 0.1). While for MIZ, these changes involved: volume of Ag-NPs (500.0 μL ± 5), Britton–Robinson buffer pH (9 ± 0.2), and volume of buffer (1 mL ± 0.1).

Finally, the selectivity was investigated by studying the ability of the proposed method to detect the studied drugs in their commercial preparations, spiked human plasma, and in the presence of other antifungal and common co-administered drugs.

### Assay of tablets

Ten tablets of Astranida^®^ (500 mg ONZ/tablet) were weighed and ground. An equivalent amount of powder to 10.98 mg of ONZ was mixed with 30 mL of methanol and sonicated for about 20 min. The mixture was then filtered, transferred to 50 mL volumetric flask and completed with methanol to the mark. Working solutions were prepared by dilution with double distilled water using 10 mL volumetric flasks. The procedure described under “Spectrofluorimetric measurements” section was then followed and the corresponding regression equation was used to determine the nominal contents of tablets.

### Analysis of cream and gel dosage forms

An accurately weighed amounts of Daktarin^®^ cream (20 mg MIZ/1 g) and Daktarin^®^ oral gel (20 mg miconazole/1 g) equivalent to 23.95 mg and 20.81 mg of miconazole nitrate and miconazole, respectively, were added separately to 40 mL of methanol and sonicated for 20 min at 60 °C. The resulting solutions were cooled for 2 min to solidify the base, then filtered, washed and completed to 50 mL with methanol using 50 mL volumetric flask. Different concentrations within the concentration range of MIZ were prepared after dilution with double distilled water. The procedure described under “Spectrofluorimetric measurements” section was then followed and the corresponding regression equation was used to determine the nominal contents of the cream and gel dosage forms.

### Procedure for human plasma

Into a series of 15-mL Falcon tubes, 1 mL aliquots of ONZ standard solution were added to 1 mL human plasma and vortex mixed for 2 min. Then 0.5 mL of Ag-NPs and 2 mL Britton–Robinson buffer (pH 4) were added. Protein precipitation was achieved by completing to 5 mL with methanol. The final concentration was in the range of 20.0–40.0 μM ONZ. The solution was then vortex mixed for 2 min and centrifuged at 6000 rpm for 20 min. The supernatant (3.5 mL) was filtered using syringe filter 0.45 μm and fluorescence measurements were performed. Blank plasma was prepared in a similar way. The concentration measurements were performed as described under “Spectrofluorimetric measurements” section and the corresponding regression equation was derived. All experiments were performed in accordance with the Institutional Ethics Approval of the relevant University Committee (Committee of Research Ethics in the Faculty of Pharmacy, Kafrelsheikh University, Kafrelsheikh, Egypt).

### Fluorescence quantum yield measurements

The quantum yield of Ag-NPs was calculated using the following Eq. ^52^:

$$\Phi_{Ag - NPs} = \Phi_{St} \times \left. {\left( {\frac{{F_{Ag - NPs} }}{{F_{St} }}} \right.} \right) \times \left. {\left( {\frac{{A_{St} }}{{A_{Ag - NPs} }}} \right.} \right) \times \left. {\left( {\frac{{\eta_{Ag - NPs} }}{{\eta_{St} }}} \right.} \right)^{2}$$

where Φ represents the quantum yield, F is the integrated fluorescence intensity of Ag-NPs and the standard (St) after excitation at 280 nm, A is the absorbance at 280 nm, and η is the refractive index of solvent (η = 1.33 for both solvents). 2-amino pyridine in 0.1 M H_2_SO_4_ was used as a standard with Φ_St_ equals 0.6. The absorbance (A_St_ and A_Ag-NPs_) was kept less than 0.1 in order to minimize the absorption effect.

### Ethics declarations

All experiments were performed in accordance with relevant guidelines and regulations and this work was approved by the Committee of Research Ethics in the Faculty of Pharmacy, Kafrelsheikh University, Kafrelsheikh, Egypt.

### Informed consent

A waiver for the informed consent for the current study was obtained from the Committee of Research Ethics in the Faculty of Pharmacy, Kafrelsheikh University, Kafrelsheikh, Egypt.

## Results and discussion

### Characterization of Ag-NPs

In this study, the green synthesis of Ag-NPs was carried out using *Piper cubeba* seed extract for the first time (Fig. 2). Under UV lamp, the synthesized Ag-NPs solution showed strong green fluorescence (Fig. S1). They were characterized using UV–visible spectrophotometry, spectrofluorimetry, DLS, EDX, FT-IR spectrometry, and HRTEM.

**Figure 3:**
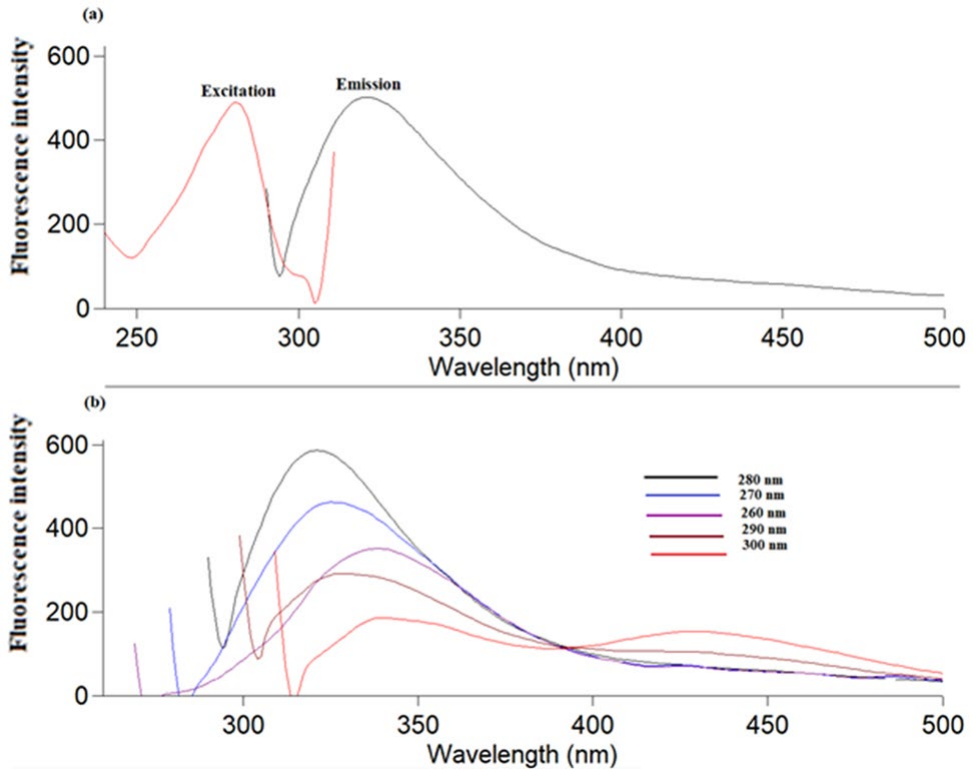
(**a**) Fluorescence emission spectra of Ag-NPs at 320 nm after excitation at 280 nm, (**b**) Fluorescence emission spectra of the Ag-NPs at different excitation wavelengths (260–300 nm).

Figure S2 shows the UV–visible absorption spectra of each of AgNO_3_ and Ag-NPs. As observed, Ag-NPs showed maximum UV absorption peak at 390 nm^53^. Fluorescence spectra of Ag-NPs are presented in Fig. 3a, as Ag-NPs were excited at 280 nm, the maximum emission peak appeared at 320 nm. The emission showed excitation wavelength dependency. For detecting the optimum excitation wavelength (λ_ex_) that result in the optimal emission, λ_ex_ was changed from 260 to 300 nm and 280 nm was found to be the optimum λ_ex_ (Fig. 3b).

**Figure 4:**
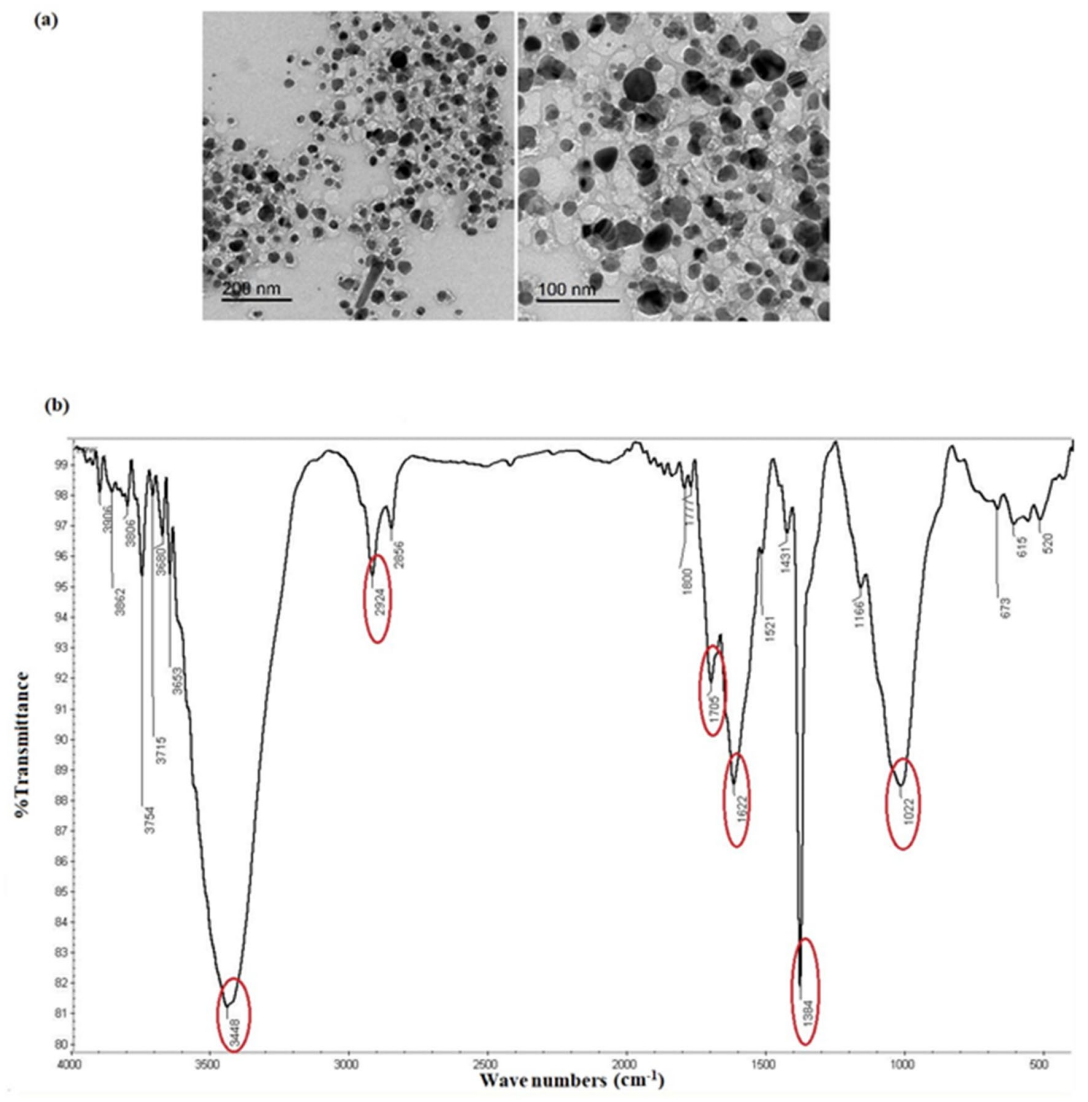
(**a**) HRTEM images of Ag-NPs, (**b**) FT-IR spectra of Ag-NPs.

The morphology of Ag-NPs was examined using HRTEM which revealed that Ag-NPs were well separated with spherical shape (Fig. 4a). As well, DLS was used to measure the particle size distribution of Ag-NPs, which had a main average size of about 88 nm and polydispersity of 0.202 (Fig. S3a). EDX spectroscopy was also performed to investigate the elemental composition of Ag-NPs, which showed absorption peaks of silver at 3 kV confirming the formation of Ag-NPs (Fig. S3b). Other peaks also appeared as C, O, Cl, and K that could be due to their presence in the *Piper cubeba* extract.

FT-IR was used to study the surface functional groups of Ag-NPs. The obtained spectra showed different functional groups including N–H (3448 cm^−1^), C–H (2924 cm^−1^), C=O (1705 cm^−1^), C=C (1622 cm^−1^), C–N (1384 cm^−1^), and C–O (1022 cm^−1^)^54^ as shown in Fig. 4b.

The quantum yield of Ag-NPs was also evaluated using 2-amino pyridine as a standard and they showed a good quantum yield of about 0.45.

### Antimicrobial activity of Ag-NPs

**Figure 5:**
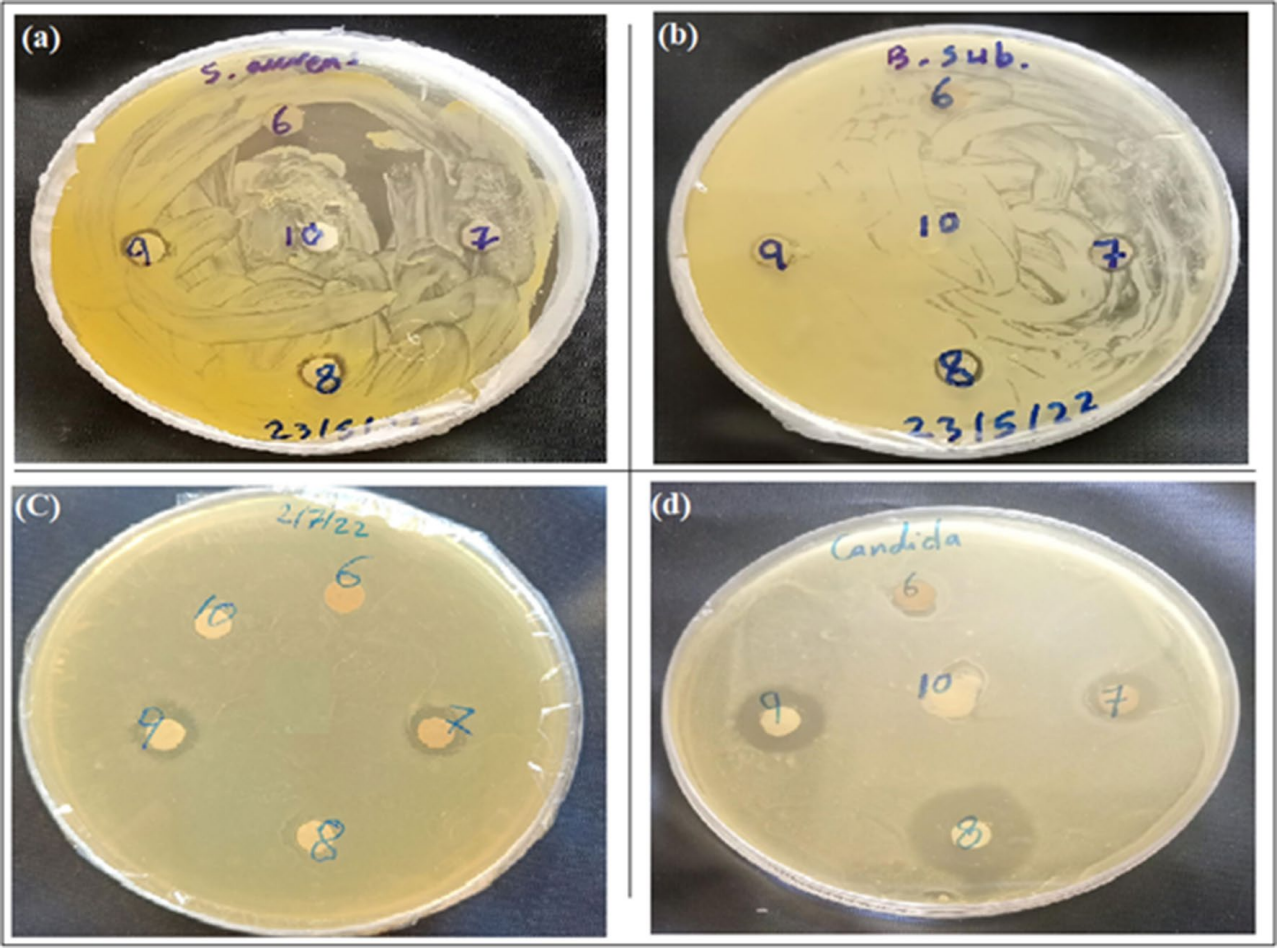
Antimicrobial activity of *Piper cubeba* extract (6), Ag-NPs (50.0 µg/mL) (7), MIZ (1.0 mM) (8), AgNO_3_ (1.0 mM) (9), and ONZ (1.0 mM) (10) against (**a**) *S. aureus*, (**b**) *B. subtilis*, (**c**) *E. coli*, and (**d**) *C. albicans*.

The antimicrobial activity of Ag-NPs was investigated against various pathogenic organisms including, gram positive bacteria (*B. subtilis, S. aureus*), gram negative bacteria (*E. coli*), and *Candida albicans* fungi. Upon comparing Ag-NPs with ONZ and *Piper cubeba* extract, Ag-NPs were found to have greater antimicrobial activity against *candida albicans* (11.21 mm), *B. subtilis* (9.12 mm), *S. aureus* (8.86 mm), and *E. coli* (7.41 mm) in contrast to the extract and ONZ that have less antifungal activity (*Piper cubeba* extract, 9.25 mm and ONZ, 9.46 mm) and no antibacterial action. In addition, the prepared Ag-NPs were also compared with AgNO_3_ and MIZ, and it was found that, all the four pathogens were susceptible for MIZ and AgNO_3_ except for *B. subtilis* which was more susceptible for Ag-NPs (Table 1, Fig. 5).

**Table 1:** Antimicrobial activity of Ag-NPs using agar disc diffusion method.

	Inhibition zone diameter (mm)			
	*Candida albicans*	*S. aureus*	*B. subtilis*	*E. coli*
*Piper cubeba* extract	9.25	0.00	0.00	0.00
Ag-NPs (50.0 µg/mL)	11.21	8.86	9.12	7.41
AgNO_3_ (1.0 mM)	12.31	9.38	8.59	7.09
ONZ (1.0 mM)	9.46	0.00	0.00	0.00
MIZ (1.0 mM)	15.14	9.42	8.99	9.91

### Mechanism of fluorescence quenching

**Figure 6:**
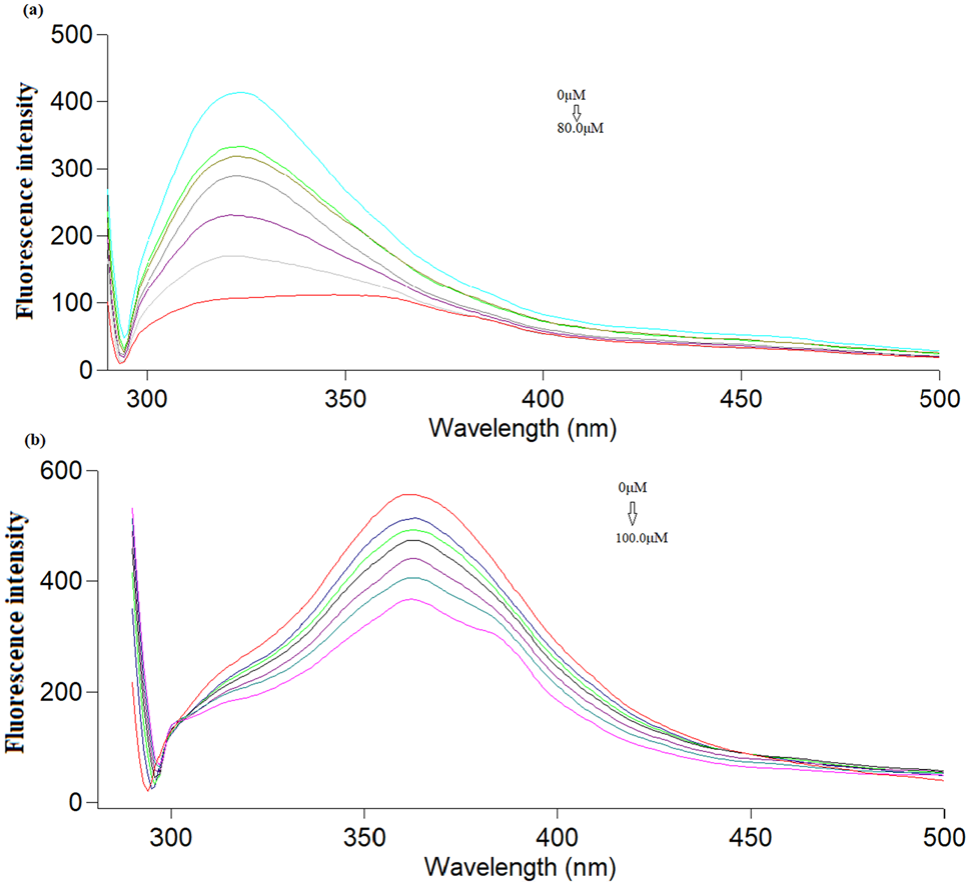
Fluorescence emission spectra of Ag-NPs upon the addition of different concentrations of (**a**) ONZ (from top to bottom: 0, 5.0, 10.0, 20.0, 40.0, 60.0, 80.0 μM), (**b**) MIZ (from top to bottom: 0, 20.0, 30.0, 40.0, 60.0, 80.0, 100.0 μM).

Figure 6 shows the fluorescence spectra of Ag-NPs in presence of each of ONZ and MIZ with different concentrations. The fluorescence quenching of Ag-NPs increases quantitatively by increasing the concentration of the studied drugs.

**Figure 7:**
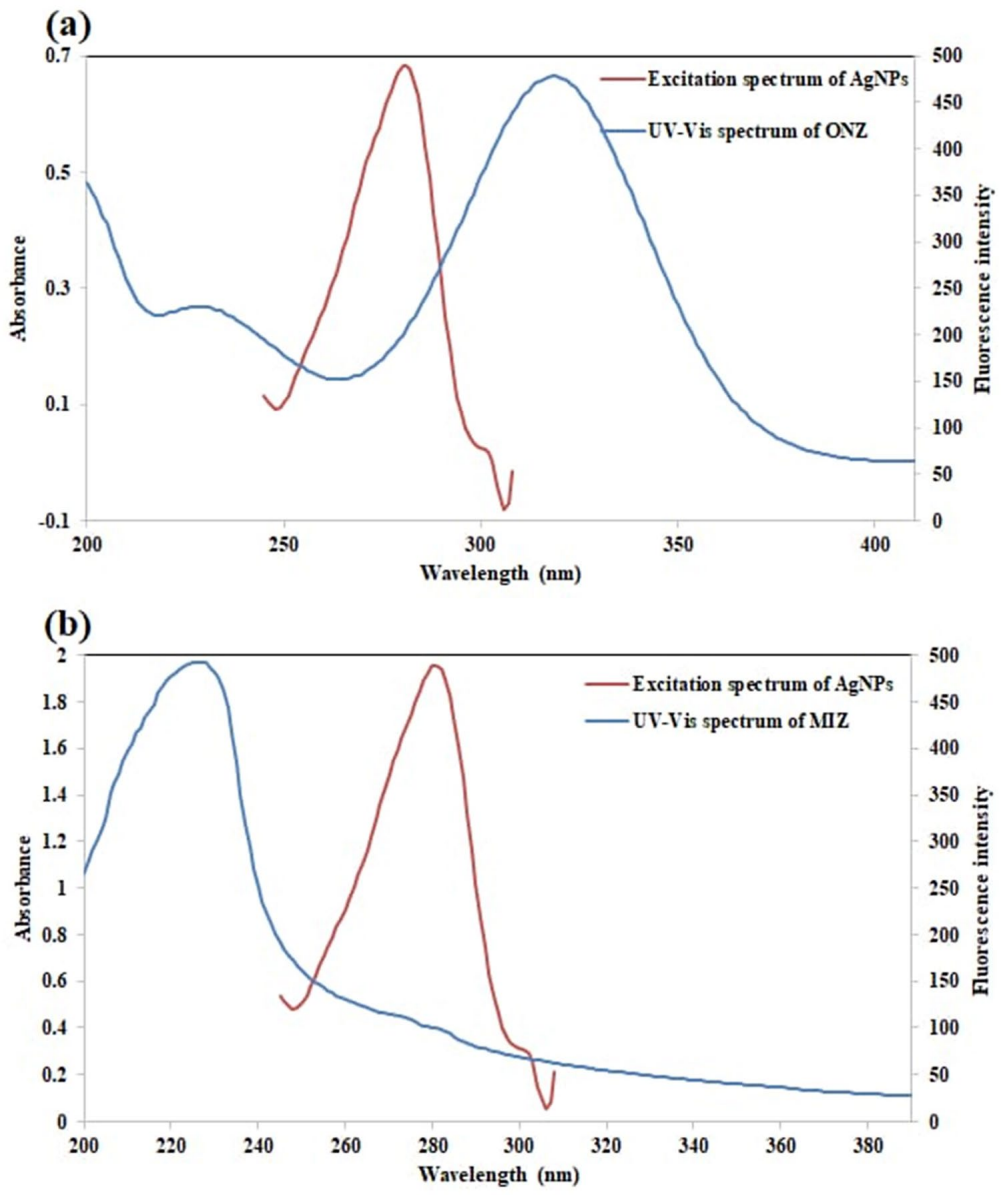
(**a**) A co-plot showing the overlapping between the fluorescence excitation spectrum of Ag-NPs and the UV–Vis absorption spectrum of ONZ, (**b**) A co-plot showing the absence of overlapping between the fluorescence excitation spectrum of Ag-NPs and the UV–Vis absorption spectrum of MIZ.

Generally, quenching of fluorescence has different mechanisms, including inner filter effect (IFE), dynamic quenching, and static quenching^55^. Since there was an overlapping between the excitation spectra of Ag-NPs and the UV–visible absorption spectra of ONZ, IFE could occur (Fig. 7a). In contrary, no overlapping occurred between MIZ absorption spectra and Ag-NPs excitation spectra, and that resulted in excluding the IFE in this case (Fig. 7b).

For prospective IFE, the intensity of the fluorescence of Ag-NPs was corrected following the addition of ONZ in increasing concentrations using Eq. (3):

3 $${\varvec{F}}_{corr} = {\varvec{F}}_{obs} \times 10^{{\left( {\frac{{A_{{{\text{ex}}}} + A_{{{\text{em}}}} }}{2}} \right)}}$$

where **F**_**corr**_ and **F**_**obs**_ are the fluorescence intensity corrected and observed after excluding the IFE from the **F**_**obs**_., **A**_**ex**_ and **A**_**em**_ refer to the drug absorbance at the excitation and emission wavelengths, respectively.

The suppressed efficiency (**%E**) was calculated for both the corrected and observed fluorescence according to Eq. (4):

4 $$\% {\varvec{E}} = \left[ {1 - \left( {\frac{{\varvec{F}}}{{{\varvec{F}}_{0} }}} \right)} \right] \times 100$$

where **%E** is the suppressed efficiency, **F** is the **F**_**corr**_ or **F**_**obs**_, **F**_**0**_ is the blank fluorescence intensity.

**Figure 8:**
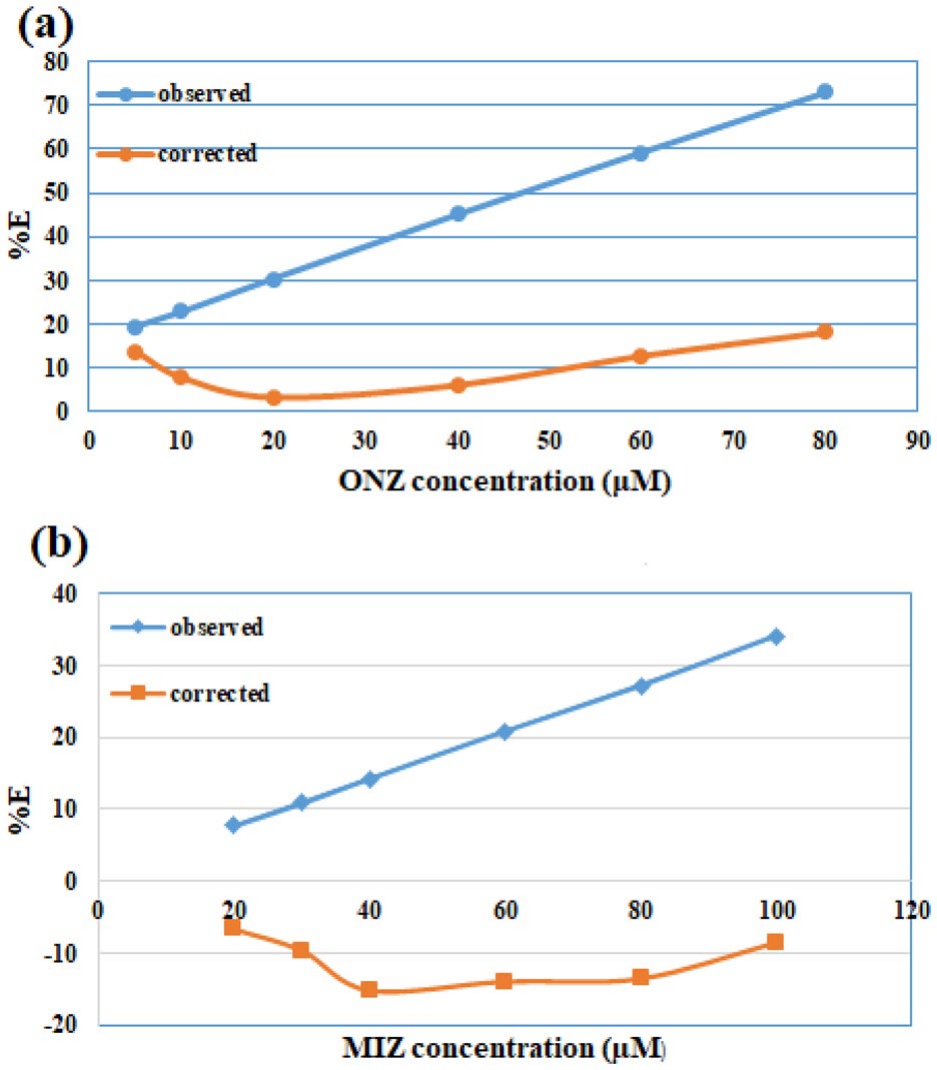
%E of observed and corrected fluorescence of Ag-NPs after the addition of various concentrations of (**a**) ONZ (5.0, 10.0, 20.0, 40.0, 60.0, 80.0 μM), (**b**) MIZ (20.0, 30.0, 40.0, 60.0, 80.0, 100.0 μM).

The **%E** of both corrected and observed fluorescence intensities of Ag-NPs were separately plotted against ONZ and MIZ concentrations in µM. From Fig. 8 we can conclude that the IFE plays an important role in the quenching of Ag-NPs by ONZ, while for MIZ there was no effect.

In addition to the IFE, other mechanisms might occur like static or dynamic quenching. The static quenching occurs due to the ground state complex formation. While, in dynamic quenching, the quencher (ONZ or MIZ) diffuses in the excited state to the fluorophore and contacts with it^56^. Another difference between the two prescribed mechanisms is the dependency on temperature. In the dynamic quenching, increasing the temperature increases the Stern–Volmer constant, while decreases it in the static one^57^. The possible quenching mechanisms were investigated using Stern–Volmer Eq. (5):

5 $$\frac{{F_{0} }}{F} = 1 + K_{sv} \left[ Q \right]$$

where (F_0_) and (F) are the fluorescence intensities before and after adding the drug, K_SV_ is the Stern–Volmer (S–V) constant, [Q] is the concentration of quencher^56^.

**Figure 9:**
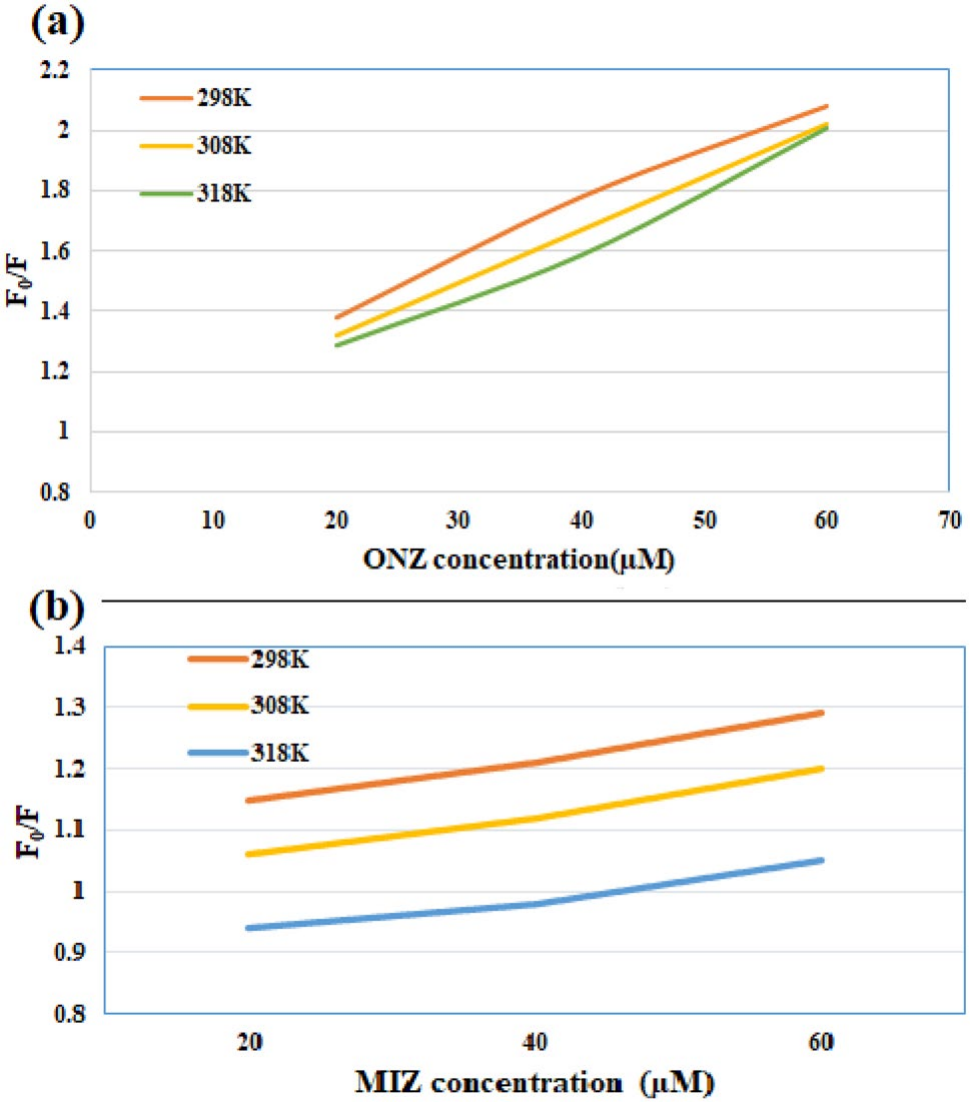
Stern–Volmer plots for the quenching of Ag-NPs fluorescence at three different temperature settings (298, 308, and 318 K) by different concentrations of (**a**) ONZ (20.0, 40.0, 60.0 μM), (**b**) MIZ (20.0, 40.0, 60.0 μM).

The proposed method was performed at different temperature settings (298, 308, 318 K) and S–V equation was adopted where F^o^/F was plotted against the concentration of each drug in µM (Fig. 9). For ONZ, the K_SV_ values were 18.2 × 10^3^, 17.05 × 10^3^, and 16.65 × 10^3^, while for MIZ the K_SV_ values were 4.65 × 10^3^, 3.3 × 10^3^, and 0.95 × 10^3^ at 298, 308, 318 K, respectively. As a result, in addition to the previously discussed IFE, the mechanism of quenching for both ONZ and MIZ is the static quenching since K_SV_ decreased by increasing the temperature^58–60^.

### Optimization of experimental parameters

#### Effect of temperature on Ag-NPs

In order to confirm the maximum formation of Ag-NPs, the effect of temperature on Ag-NPs synthesis was studied by heating the mixture at different temperature settings in the range of 30–90 °C. It was found that the absorbance (λ_max_ = 390 nm) increased by increasing the temperature, which indicated the maximum formation of Ag-NPs (Fig. S4). This was supported by the change of the color into dark brown and also was compatible with the results of most of the reported studies that investigated the effect of temperature on Ag-NPs^61,62^. Accordingly, 90 °C was used as the optimal temperature for the synthesis of Ag-NPs.

#### Effect of pH and buffer volume

Upon studying the effect of pH and buffer volume, Britton–Robinson buffers with pH 4 and pH 9 were found to result in a subsequent increase in the fluorescence quenching of Ag-NPs by ONZ and MIZ, respectively (Fig. S5a). In addition, the optimum buffer volumes were 2 mL and 1 mL for ONZ and MIZ, respectively (Fig. S5b).

#### Effect of incubation time

It was found that quenching of the fluorescence intensity of Ag-NPs by both drugs was stable for about 10 min then the quenching effect slightly decreased with increasing the incubation time (Fig. S5c).

#### Effect of temperature

Increasing the temperature was found to result in a significant decrease in the fluorescence quenching with the studied drugs (Fig. S5d). As a result, the spectrofluorimetric measurements were performed at room temperature.

### Method validation

The proposed method was validated according to ICHQ2 (R1) guidelines^50^.

#### Linearity and range

The calibration curve was obtained by plotting fluorescence quenching (F_0 _− F) versus different drug concentrations in μM which was linear over the range of (5.0–80.0 μM) and (20.0–100.0 μM) for ONZ and MIZ, respectively. The correlation coefficient (r) for both drugs was 0.9999. Linear regression analysis was represented using the following equations:

6 $$\left( {{\text{F}}_{0} - {\text{F}}} \right) = 2.9667{\text{\,C}} + 66.05\;\;\;\; \left( {{\text{r}} = 0.9999} \right){\text{ for ONZ}}$$

7 $$\left( {{\text{F}}_{0} - {\text{F}}} \right) = 1.8221{\text{\,C}} + 6.4803\;\;\;\;\left( {{\text{r }} = 0.9999} \right){\text{ for MIZ}}$$

where F_0_ is the native fluorescence intensity of Ag-NPs, F is the Ag-NPs fluorescence intensity in presence of ONZ or MIZ, C is the concentration of the studied drugs in μM, and r is the correlation coefficient. The developed method’s analytical performance data was summarized in Table 2.

**Table 2:** Analytical performance data of the proposed method.

Parameters	ONZ	MIZ
Linear concentration range (µM)	5.0–80.0	20.0–100.0
LOD (µM)	0.35	1.43
LOQ (µM)	1.06	4.35
Regression equation	(F_0 _− F) = 2.9667C + 66.05	(F_0 _− F) = 1.8221C + 6.4803
Correlation coefficient (r)	0.9999	0.9999
S.D. of the residuals, S_y/x_	1.11	0.88
S.D. of the intercept, S_a_	0.31	0.79
S.D. of the slope, S_b_	0.02	0.01
Percentage relative standard deviation (% RSD)	1.93	0.82
Percentage relative error (% Error)	0.78	0.34

#### LOD and LOQ

LOD and LOQ are a measure of method sensitivity that were 0.35 and 1.06 μM, respectively for ONZ and 1.43 and 4.35 μM, respectively for MIZ. The measured LOD and LOQ values proved that the proposed method is adequately sensitive (Table 2).

#### Accuracy and precision

Upon comparing the results obtained by the proposed method with those obtained by the reported methods^29,42^, the analytical data was statically analyzed using both Variance ratio F-test and Student t-test which showed that there was no marked difference between the precision and accuracy of the proposed and reported methods (Table 3). The intra and inter-day precisions were also evaluated and the investigated drugs showed acceptable % error and %RSD, as presented in Table S1.

**Table 3:** Application of the proposed method for the determination of the studied drugs in raw materials.

Parameter	ONZ			MIZ		
	Conc. taken (μM)	Conc. found (μM)	% Recovery^a^	Conc. taken (μM)	Conc. found (μM)	% Recovery^a^
	5.0	4.87	97.44	20.0	20.23	101.16
	10.0	9.79	97.99	30.0	29.73	99.11
	20.0	19.87	99.36	40.0	40.11	100.27
	40.0	40.69	101.74	60.0	60.21	100.35
	60.0	60.17	100.29	80.0	79.26	99.07
	80.0	79.59	99.48	100.0	100.46	100.46
Mean			99.13			100.07
± SD			1.92			0.82
% RSD			1.93			0.82
% Error			0.78			0.34

	Comparison method^29^			Comparison method^42^		
Mean ± SD	98.57 ± 1.33			99.61 ± 0.51		
N^c^	3			3		
t value	0.77 (2.36)^b^			0.87 (2.36)^b^		
F value	1.37 (19.30)^b^			2.59 (19.30)^b^		

^a^Mean of three determinations.

^b^Values in parenthesis are the tabulated t and F values at *p* = 0.05^51^.

^c^Number of samples.

#### Robustness

As described under “Experimental” section, the robustness of the developed method was studied to find out if small changes in the experimental parameters could influence the quenching of the fluorescence intensities of Ag-NPs by ONZ and MIZ. The obtained results indicated that, small changes in experimental conditions did not significantly affect the quenching of the Ag-NPs fluorescence intensity by both drugs, as shown in Table S2.

#### Selectivity

The selectivity of the proposed method for determining ONZ and MIZ was verified in their commercial dosage forms with low %RSD (less than 1.59%) and high % recovery (98.0–101.04%) without any interference from the existed excipients. The possible interfering excipients as lactose, maltose, mannitol, dextrin, and citric acid were studied in details and confirmed the high selectivity of the method, since they almost did not affect the fluorescence intensity of the Ag-NPs (Fig. S6). Similarly, the method selectivity was proved, by its ability to detect the studied drugs in the presence of different metal ions like Na^+^, K^+^, Ca^+2^, Mg^+2^, and Ba^+2^ without any interference (Fig. S7). The proposed method was also able to determine ONZ and MIZ in the presence of other antifungal and common co-administered drugs such as clotrimazole, ketoconazole, terconazole, ciprofloxacin, ofloxacin, and hydrocortisone sodium succinate. The tolerance limit of the drugs was determined as the concentration that result in 2% or less relative error, which was 25.0 µM for clotrimazole, 10.0 µM for both ketoconazole and terconazole, 1.0 µM for both ciprofloxacin and ofloxacin, and 25.0 µM for hydrocortisone sodium succinate^60^. In addition, the method could determine ONZ in spiked human plasma with low %RSD and high % recoveries demonstrating the absence of interference from plasma components. Accordingly, the developed method showed excellent selectivity for determination of ONZ and MIZ without any interference.

### Method applications

#### Analysis of ONZ and MIZ in commercial dosage forms

The studied drugs were successfully determined in their commercial dosage forms including, Astranida^®^ tablets for ONZ, Daktarin^®^ cream, and Daktarin^®^ oral gel for MIZ with high % recovery (98.0–101.04%) and low %RSD values (less than 1.59%). Table 4 shows that the results of the proposed method were in an acceptable agreement with those obtained by the reported methods^29,42^. Statistical analysis of the resultant data by Variance ratio F-test and Student t-test indicated that the method has good accuracy and precision, respectively.

**Table 4:** Application of the proposed method for the determination of the studied drugs in commercial dosage forms.

Parameter	Astranida® film-coated tablets (ONZ 500 mg / tablet)					
	Conc. Taken (μM)		Conc. Found (μM)		% recovery^a^	
	40.0		39.33		98.33	
	50.0		49.14		98.28	
	60.0		60.62		101.04	
Mean					99.22	
± SD					1.58	
% RSD					1.59	

	Comparison method ^29^					
Mean ± SD	100.24 ± 0.95					
N^c^	3					
t-value	0.302 (2.77)^b^					
F-value	2.66 (19.00)^b^					

Parameter	Daktarin® cream (MIZ 20 mg / 1 g)			Daktarin® oral gel (Miconazole 20 mg / 1 g)		
	Conc. taken (μM)	Conc. found (μM)	% recovery^a^	Conc. taken (μM)	Conc. found (μM)	% Recovery^a^
	20.0	19.66	98.28	20.0	20.11	100.56
	40.0	39.2	98.0	40.0	39.98	99.96
	60.0	59.17	98.62	60.0	59.53	99.21
Mean			98.63			99.91
± SD			0.71			0.68
% RSD			0.72			0.68

	Comparison method ^42^			Comparison method ^42^		
Mean ± SD	98.08 ± 0.58			99.32 ± 1.83		
N^c^	3			3		
t-value	0.57 (2.78)^b^			0.52 (2.78)^b^		
F-value	3.43 (19.0)^b^			7.33 (19.0)^b^		

^a^Mean of three determinations.

^b^Values in parenthesis are the tabulated t- and F- values at p = 0.05 ^51^.

^c^Number of samples.

#### Determination of ONZ in human plasma

The selectivity and high sensitivity of the proposed analytical method allowed its application to analyze and/or detect ONZ in spiked human plasma, since the mean maximum plasma concentration of ONZ was 10.9 μg/ml (49.63 µM) when a dose of 750 mg was administered^63^. Linear relationship was found in plasma samples spiked with ONZ after plotting the quenching of fluorescence (F_0 _− F) against the concentrations of ONZ in µM. It was demonstrated that the method has sufficient ability to analyze the studied drug with high mean percentage recoveries and low SD values (100.08% ± 1.75), indicating that endogenous plasma components did not interfere, which pointed out to a negligible matrix effect (Table 5, Fig. S8). Linear regression analysis is represented by the following Eq. (8):

8 $$\left( {{\text{F}}_{0} - {\text{F}}} \right) = 10.32{\text{\,C}}{-}111.12 \;\;\;\;\left( {{\text{r }} = \, 0.999} \right)$$

**Table 5:** Application of the proposed method for the determination of ONZ in spiked human plasma.

Parameter	Conc. taken (μM)	Conc. found (μM)	% Recovery^a^
	20.0	20.29	101.43
	30.0	29.43	98.10
	40.0	40.29	100.71
Mean			100.08
± SD			1.75
% RSD			1.75
% Error			1.01

^a^Mean of three determinations.

### Comparison with other methods

In comparison to the other reported methods, the proposed method provides a simple, fast, and an ecofriendly spectrofluorimetric approach for the estimation of ONZ and MIZ instead of the expensive, complicated, and tedious HPLC, capillary electrophoresis, and other chromatographic techniques. The proposed method is the first spectrofluorimetric method for the determination of MIZ and the first one used Ag-NPs as a fluorescent probe for the estimation of ONZ without the need for any pre-derivatization steps. Since the studied drugs don't exhibit native fluorescent properties, the importance of the proposed study is magnified. Compared to the reported spectrofluorimetric method for ONZ^30^, the proposed method is almost as sensitive as the reported one, which was not green, time consuming, and more complicated.

## Conclusion

The current study provides a green, simple, rapid, and economic spectrofluorimetric method for the estimation of ONZ and MIZ. This method is the first spectrofluorimetric one for the determination of the studied drugs using Ag-NPs without the need for any pre-derivatization steps. Since the studied drugs don't exhibit native fluorescent properties, the importance of the proposed study is magnified. The developed method depends on the ecofriendly synthesis of Ag-NPs using *Piper cubeba* seed extract. The produced Ag-NPs were characterized using UV–Vis spectroscopy, spectrofluorimetry, FTIR, DLS, EDX, and HRTEM. The synthesized Ag-NPs showed a strong green fluorescence, which was quantitatively quenched by the studied drugs. Antimicrobial activity of Ag-NPs was examined using the disc diffusion method. The synthesized nanoparticles exhibited marked antimicrobial activity against various strains of gram negative and gram positive bacteria in addition to *Candida albicans* fungi. Therefore, the proposed method may hold potential applications in the antimicrobial therapy and related mechanism research. The developed method was applied for determining the studied drugs in commercial dosage forms with high % recoveries and low %RSD values. The proposed method was also used for the sensitive detection and determination of ONZ in the spiked human plasma.

## Supplementary Information

Supplementary Information.

## Data Availability

The datasets generated and/or analyzed during the current study are available from the corresponding author on reasonable request.

## Abbreviations

Ag-NPs: Silver nanoparticles
DLS: Dynamic light scattering
EDX: Energy-dispersive X-ray
FTIR: Fourier transform infrared
HPLC: High performance liquid chromatography
HPTLC: High performance thin layer chromatography
HRTEM: High-resolution transmission electron microscope
IFE: Inner filter effect
LOD: Limit of detection
LOQ: Limit of quantitation
MIZ: Miconazole nitrate
ONZ: Ornidazole
RSD: Relative standard deviation
TLC: Thin layer chromatography

## References

[1] Nahak G, Sahu R (2011). Phytochemical evaluation and antioxidant activity of *Piper cubeba* and *Piper nigrum*. J. Appl. Pharm. Sci..

[2] Yu S-J, Yin Y-G, Liu J-F (2013). Silver nanoparticles in the environment. Environ. Sci. Process. Impacts.

[3] Heinemann MG, Rosa CH, Rosa GR, Dias D (2021). Biogenic synthesis of gold and silver nanoparticles used in environmental applications: A review. Trends. Environ. Anal. Chem..

[4] Haider A, Kang I-K (2015). Preparation of silver nanoparticles and their industrial and biomedical applications: A comprehensive review. Adv. Mater. Sci. Eng..

[5] Yaqoob AA, Umar K, Ibrahim MNM (2020). Silver nanoparticles: Various methods of synthesis, size affecting factors and their potential applications–a review. Appl. Nanosci..

[6] Burdușel A-C (2018). Biomedical applications of silver nanoparticles: An up-to-date overview. Nanomaterials.

[7] McNamara K, Tofail SA (2017). Nanoparticles in biomedical applications. Adv. Phys..

[8] Jouyban A, Rahimpour E (2020). Optical sensors based on silver nanoparticles for determination of pharmaceuticals: An overview of advances in the last decade. Talanta.

[9] Mathur P, Jha S, Ramteke S, Jain N (2018). Pharmaceutical aspects of silver nanoparticles. Artificells Nanomed. Biotechnol..

[10] Ahmed S, Ahmad M, Swami BL, Ikram S (2016). A review on plants extract mediated synthesis of silver nanoparticles for antimicrobial applications: A green expertise. J. Adv. Res..

[11] Mittal AK, Chisti Y, Banerjee UC (2013). Synthesis of metallic nanoparticles using plant extracts. Biotechnol. Adv..

[12] Akintayo G (2020). IOP Conference Series: Materials Science and Engineering.

[13] Lateef A, Ojo SA, Elegbede JA (2016). The emerging roles of arthropods and their metabolites in the green synthesis of metallic nanoparticles. Nanotechnol. Rev..

[14] Sadowski Z, Maliszewska I, Grochowalska B, Polowczyk I, Kozlecki T (2008). Synthesis of silver nanoparticles using microorganisms. Mater. Sci. Poland..

[15] Alavi M (2022). Bacteria and fungi as major bio-sources to fabricate silver nanoparticles with antibacterial activities. Expert Rev. Anti-infect. Ther..

[16] Adelere, I. A. & Lateef, A. Microalgal nanobiotechnology and its applications—A brief overview. *Microb. Nanobiotechnol.* 233–255 (2021).

[17] Ahmad S (2019). Green nanotechnology: A review on green synthesis of silver nanoparticles—An ecofriendly approach. Int. J. Nanomed..

[18] Sathishkumar R (2019). Green synthesis of silver nanoparticles by bloom forming marine microalgae Trichodesmium erythraeum and its applications in antioxidant, drug-resistant bacteria, and cytotoxicity activity. J. Saudi Chem. Soc..

[19] Hasanin M, Elbahnasawy MA, Shehabeldine AM, Hashem AH (2021). Ecofriendly preparation of silver nanoparticles-based nanocomposite stabilized by polysaccharides with antibacterial, antifungal and antiviral activities. Biometals.

[20] Gupta N, Upadhyaya CP, Singh A, Abd-Elsalam KA, Prasad R (2018). Nanobiotechnology Applications in Plant Protection 247–265.

[21] Deshmukh SP, Patil S, Mullani S, Delekar S (2019). Silver nanoparticles as an effective disinfectant: A review. Mater. Sci. Eng. C.

[22] Aguda O, Lateef A (2021). Novel biosynthesis of silver nanoparticles through valorization of Parkia biglobosa fermented-seed wastewater: Antimicrobial properties and nanotextile application. Environ. Technol. Innov.

[23] Lateef A (2016). Biogenic synthesis of silver nanoparticles using a pod extract of Cola nitida: Antibacterial and antioxidant activities and application as a paint additive. J. Taibah Univ. Sci.

[24] Vazquez-Muñoz R (2019). Enhancement of antibiotics antimicrobial activity due to the silver nanoparticles impact on the cell membrane. PLoS ONE.

[25] Singh P, Mittal R, Sharma G, Singh S, Singh A (2003). Ornidazole: Comprehensive profile. Profiles Drug Subst Excip Relat Methodol..

[26] Al-Badr AA (2005). Miconazole nitrate: Comprehensive profile. Profiles Drug Subst. Excip. Relat. Methodol..

[27] Darwish KM, Salama I, Mostafa S, El-Sadek M (2012). Extractional spectrophotometric analysis of metronidazole, tinidazole, ornidazole and secnidazole bases through acid-dye complexation using bromothymol blue dye. Pak. J. Pharm. Sci..

[28] Khalile S, Elqudaby H, Ali F, Eid SM (2011). Spectrophotometric determination of ornidazole, secnidazole and tinidazole in pharmaceutical preparations based on formation of dyes. J. Pharm. Res..

[29] Rana M (2011). Spectrophotometric method development and determination of ornidazole in bulk and tablet dosage form. Int. J. Pharm Tech Res..

[30] Mehrzad-Samarin M, Faridbod F, Ganjali MR (2019). A luminescence nanosensor for ornidazole detection using graphene quantum dots entrapped in silica molecular imprinted polymer. Spectrochim. Acta A Mol. Biomol. Spectrosc..

[31] Shekar MS, Sagar JV, Narsaiah N, An R, Krishna D (2005). Validated HPLC method for the determination of ornidazole in human serum and urine. Indian J. Pharm. Sci..

[32] Bind B, Lokhande R, Munigela N, Kolhal S, Gupta A (2015). RP-HPLC method for the simultaneous determination of metronidazole, tinidazole, ornidazole, secnidazole and ofloxacin in bulk and pharmaceutical dosage form. Int. J. Pharm. Sci. Rev. Res..

[33] Shovkova OV, Klimenko LY, Shovkova ZV, Ulanova VA, Shpychak OS (2018). Application of thin layer chromatography in analysis of secnidazole, ornidazole, tinidazole and nimorazole. J. Pharm. Sci. Res..

[34] Özkan S, Şenturk Z, Biryol I (1997). Determination of ornidazole in pharmaceutical dosage forms based on reduction at an activated glassy carbon electrode. Int. J. Pharm..

[35] Turan S, Durmus Z, Kiliç E (2009). Electrochemical behavior of ornidazole and its adsorptive stripping determination in pharmaceuticals. Curr. Pharm. Anal..

[36] Ettadili F (2022). Electrochemical determination of ornidazole at silver electrode: Analytical application in human blood. Chem. Data Collect..

[37] Fonseca JL, Rivera MG, Monteagudo JG (1993). Electrochemistry of ornidazole. Anal. Lett..

[38] Phatak H, Vaidya V (2016). A rapid gas chromatography-mass spectroscopy method for simultaneous quantification of ornidazole and miconazolefrom cream formulations: Development, validation and application. Int. J. Pharm. Sci. Res..

[39] See KL, Elbashir AA, Saad B, Ali ASM, Aboul-Enein HY (2009). Simultaneous determination of ofloxacin and ornidazole in pharmaceutical preparations by capillary zone electrophoresis. Biomed. Chromatogr..

[40] Zhang L, Zhang Z, Wu K (2006). In vivo and real time determination of ornidazole and tinidazole and pharmacokinetic study by capillary electrophoresis with microdialysis. J. Pharm. Biomed. Anal..

[41] Göğer NG, Gökçen L (1999). Quantitative determination of miconazole in creams by second order derivative spectrophotometry. Anal. Lett..

[42] Kumar N (2019). Analytical method development and validation for the estimation of miconazole nitrate in bulk and marketed topical formulation. Res. J. Pharm. Technol..

[43] Ashour S, Kattan N (2010). Simultaneous determination of miconazole nitrate and metronidazole in different pharmaceutical dosage forms by gas chromatography and flame ionization detector (GC-FID). Int. J. Biomed. Sci..

[44] Korany MA, Maher HM, Galal SM, Ragab MA (2013). Development and optimization of a capillary zone electrophoresis technique for simultaneous determination of miconazole nitrate and hydrocortisone acetate in a cream pharmaceutical formulation. J. AOAC. Int..

[45] Akay C, Özkan SA, Şentürk Z, Cevheroğlu Ş (2002). Simultaneous determination of metronidazole and miconazole in pharmaceutical dosage forms by RP-HPLC. Il Farmaco.

[46] Heneedak HM, Salama I, Mostafa S, El-Sadek M (2012). HPLC and chemometric methods for the simultaneous determination of miconazole nitrate and nystatin. J. Chromatogr. Sci..

[47] Hermawan D, Sulaeman U, Istiqomah A, Aboul-Enein H (2017). IOP Conference Series: Materials Science and Engineering.

[48] Kobylińska M, Kobylińska K, Sobik B (1996). High-performance liquid chromatographic analysis for the determination of miconazole in human plasma using solid-phase extraction. J. Chromatogr. B Biomed. Sci. Appl..

[49] Patel KG, Shah PM, Shah PA, Gandhi TR (2016). Validated high-performance thin-layer chromatographic (HPTLC) method for simultaneous determination of nadifloxacin, mometasone furoate, and miconazole nitrate cream using fractional factorial design. J. Food Drug Anal..

[50] ICH, I. Q2 (R1): Validation of analytical procedures: Text and methodology. In: *International Conference on Harmonization*, Geneva (2005).

[51] Miller J, Miller JC (2018). Statistics and Chemometrics for Analytical Chemistry.

[52] Rurack K (2008). Standardization and Quality Assurance in Fluorescence Measurements I 101–145.

[53] Soto KM (2019). Fruit peels waste for the green synthesis of silver nanoparticles with antimicrobial activity against foodborne pathogens. Lwt.

[54] Smith BC (2011). Fundamentals of Fourier Transform Infrared Spectroscopy.

[55] Abd Elhaleem SM, Elsebaei F, Shalan S, Belal F (2022). Turn-off fluorescence of nitrogen and sulfur carbon quantum dots as effective fluorescent probes for determination of imatinib. Application to biological fluids. Spectrochim. Acta A Mol. Biomol. Spectrosc..

[56] Muttannavar V (2018). Effect of hydrogen bonding on ffluorescence quenching of quinolin-8-ol-analysis using negative stern–volmer plots. Int. J. Life Sci. Pharma Res..

[57] Zu F (2017). The quenching of the fluorescence of carbon dots: a review on mechanisms and applications. Michrochim. Acta..

[58] Magdy G, Hakiem AFA, Belal F, Abdel-Megied AM (2021). Green one-pot synthesis of nitrogen and sulfur co-doped carbon quantum dots as new fluorescent nanosensors for determination of salinomycin and maduramicin in food samples. Food Chem..

[59] El-Malla SF, Elshenawy EA, Hammad SF, Mansour FR (2021). N-doped carbon dots as a fluorescent nanosensor for determination of colchicine based on inner filter effect. J. Fluoresc..

[60] Magdy G, Al-enna AA, Belal F, El-Domany RA, Abdel-Megied AM (2022). Application of sulfur and nitrogen doped carbon quantum dots as sensitive fluorescent nanosensors for the determination of saxagliptin and gliclazide. R. Soc. Open Sci..

[61] Chunfa D (2018). Rapid and green synthesis of monodisperse silver nanoparticles using mulberry leaf extract. Rare Metal Mater. Eng..

[62] Khan M (2018). Plant extracts as green reductants for the synthesis of silver nanoparticles: Lessons from chemical synthesis. Dalton Trans..

[63] Schwartz D, Jeunet F (1976). Comparative pharmacokinetic studies of ornidazole and metronidazole in man. Chemotherapy.

