# Supplementary material for: Green synthesis, characterization, and antimicrobial applications of silver nanoparticles as fluorescent nanoprobes for the spectrofluorimetric determination of ornidazole and miconazole
Source: Sci Rep. 2022 Dec 10;12:21395. doi: 10.1038/s41598-022-25830-x (PMC9741645; doi:10.1038/s41598-022-25830-x)
Supplement: Supplementary file 1 — Supplementary Information. [file 41598_2022_25830_MOESM1_ESM.pdf]

Supplementary Material

Green synthesis, characterization, and antimicrobial applications of silver nanoparticles as fluorescent nanoprobes for the spectrofluorimetric determination of ornidazole and miconazole

Galal Magdy[1*], Eman Aboelkassim[1], Ramadan A. El-Domany[2], Fathalla Belal[3]

[1] Pharmaceutical Analytical Chemistry Department, Faculty of Pharmacy, Kafrelsheikh University, Kafrelsheikh, P.O. Box 33511, Egypt.

[2] Microbiology and Immunology Department, Faculty of Pharmacy, Kafrelsheikh University, Kafrelsheikh, P.O. Box 33511, Egypt.

[3] Pharmaceutical Analytical Chemistry Department, Faculty of Pharmacy, Mansoura University, Mansoura, P.O. Box 35516, Egypt.

***Corresponding author:** Galal Magdy

E-mail address: galal_magdy@pharm.kfs.edu.eg

Supplementary Figures captions

Fig. S1 Optical images of Ag-NPs under normal light (a) and UV light (b)

Fig. S2 UV-visible absorption spectrum of $AgNO_3$ (a) and Ag-NPs (b)

Fig. S3 Particle size distribution of Ag-NPs using DLS (a) and elemental analysis results of Ag-NPs using EDX (b)

Fig. S4 Effect of temperature on Ag-NPs

Fig. S5 Effect of pH (a), volume of Britton-Robinson buffer (b), incubation time (c), and temperature (d) on the relative quenched fluorescence intensity of Ag-NPs by ONZ (20.0 μM) and MIZ (50.0 μM)

Fig. S6 The response of possible interfering excipients

Fig. S7 The selectivity of Ag-NPs towards ONZ and MIZ in presence of different metal ions

Fig. S8 Fluorescence emission spectra of the Ag-NPs in spiked human plasma upon the addition of different concentrations of ONZ (from top to bottom:0.0, 20.0, 30.0, 40.0 μM)

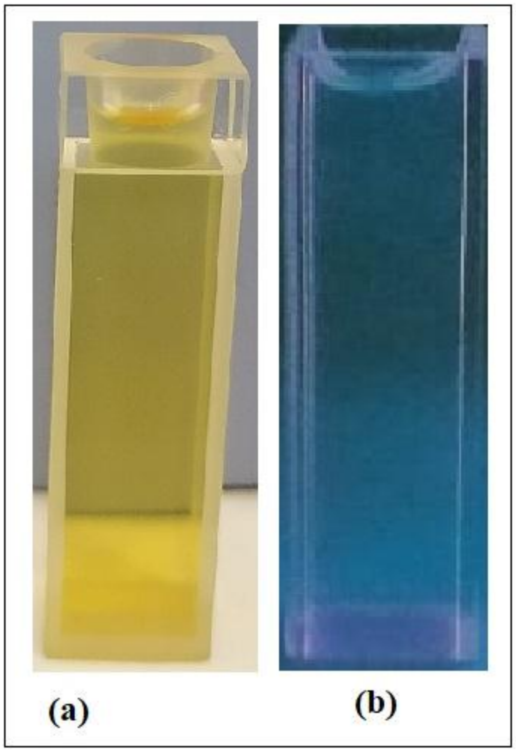
(a)
(b)

Fig. S1

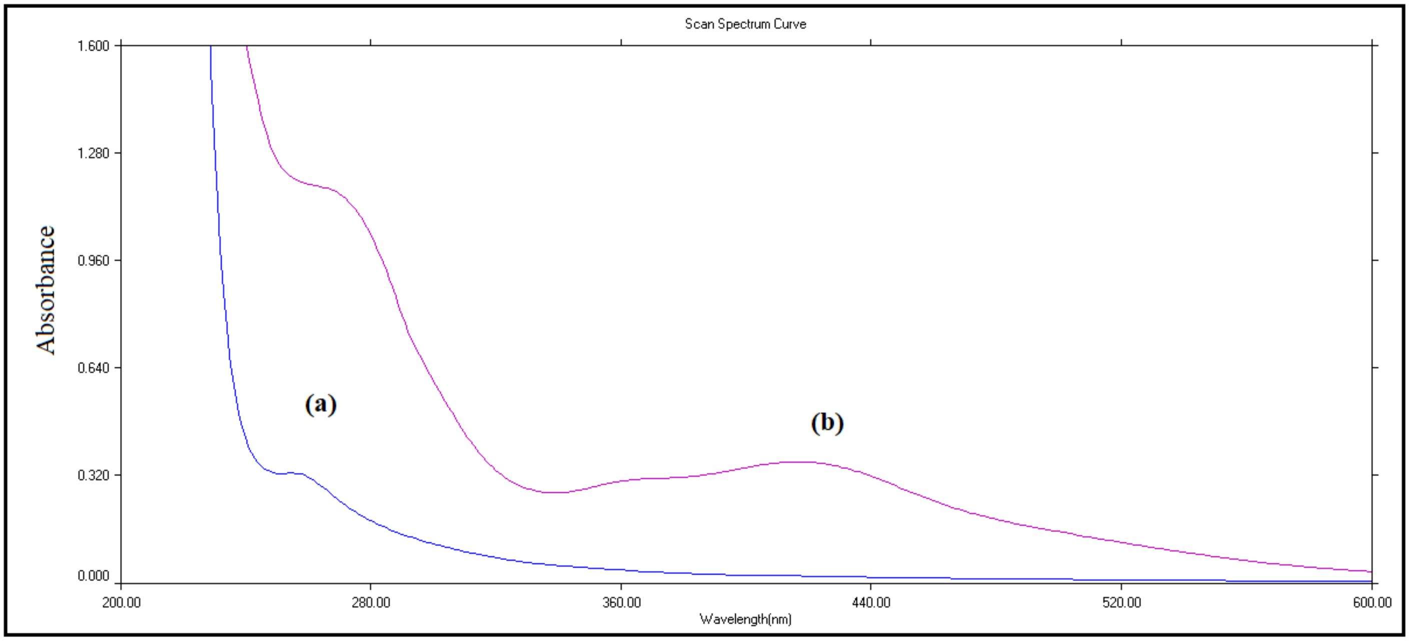
Scan Spectrum Curve
1.600
1.280
0.960
0.640
0.320
0.000
Absorbance
(a)
(b)
200.00
280.00
360.00
440.00
520.00
600.00
Wavelength(nm)

Fig. S2

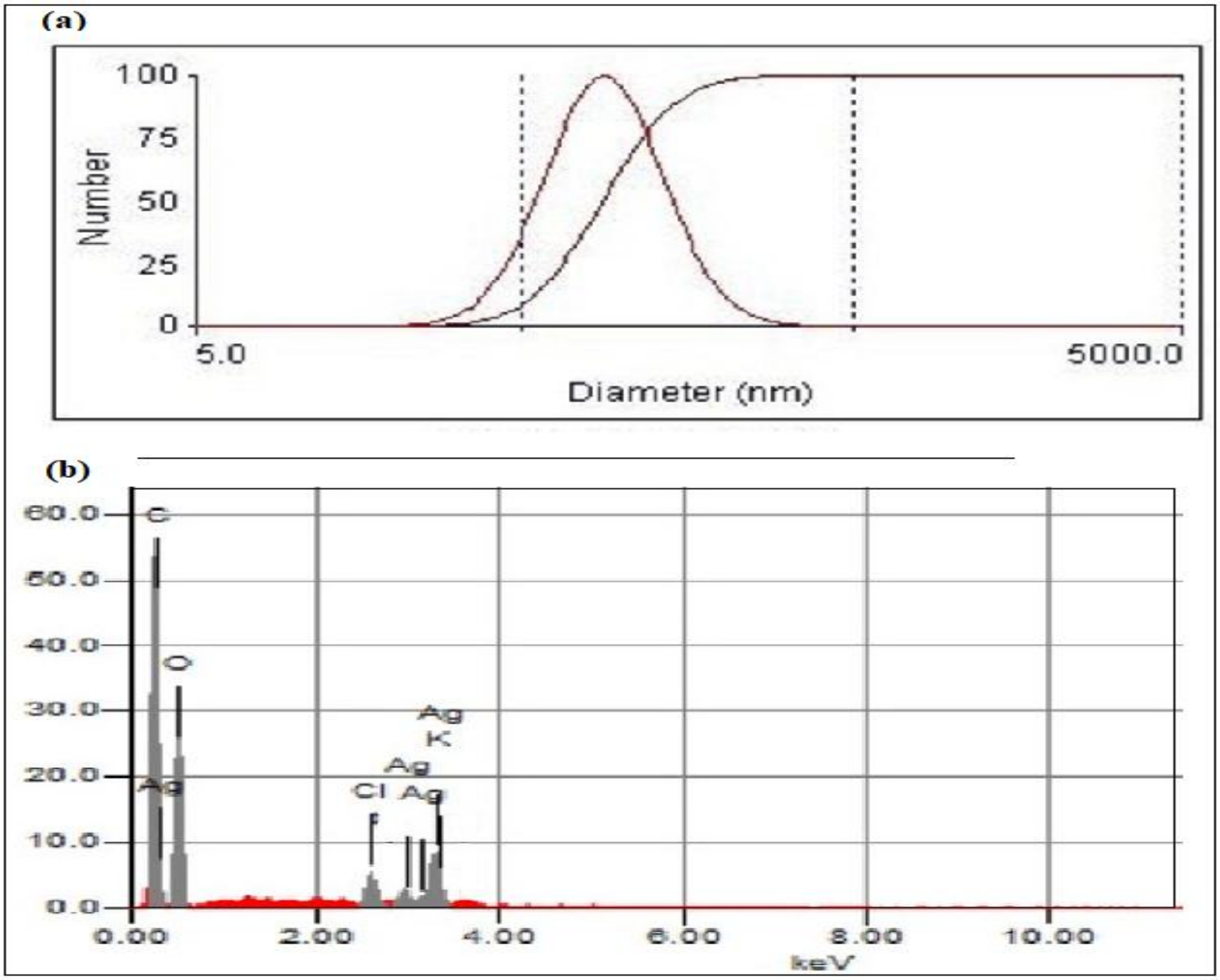
(a)
100
75
50
25
0
Number
5.0
5000.0
Diameter (nm)
(b)
60.0
50.0
40.0
30.0
20.0
10.0
0.0
C
O
Ag
Cl
Ag
Ag
K
Ag
0.00
2.00
4.00
6.00
8.00
10.00
keV

Fig. S3

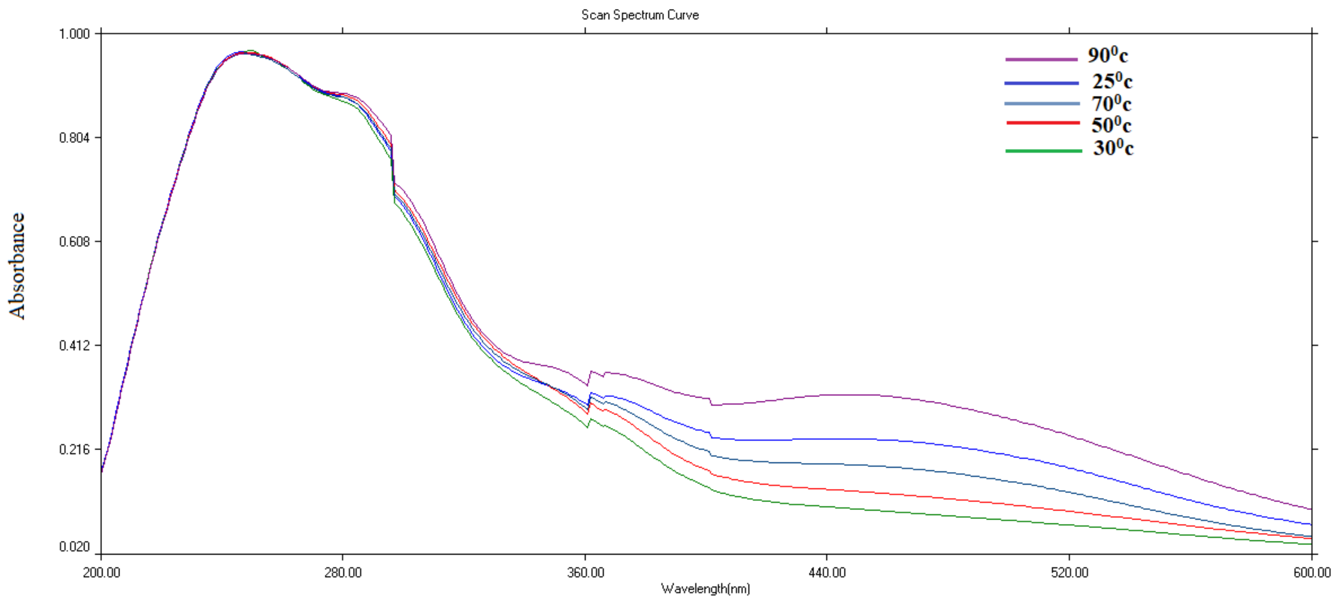
Scan Spectrum Curve
Absorbance
Wavelength(nm)
1.000
0.804
0.608
0.412
0.216
0.020
200.00
280.00
360.00
440.00
520.00
600.00
90⁰c
25⁰c
70⁰c
50⁰c
30⁰c

Fig. S4

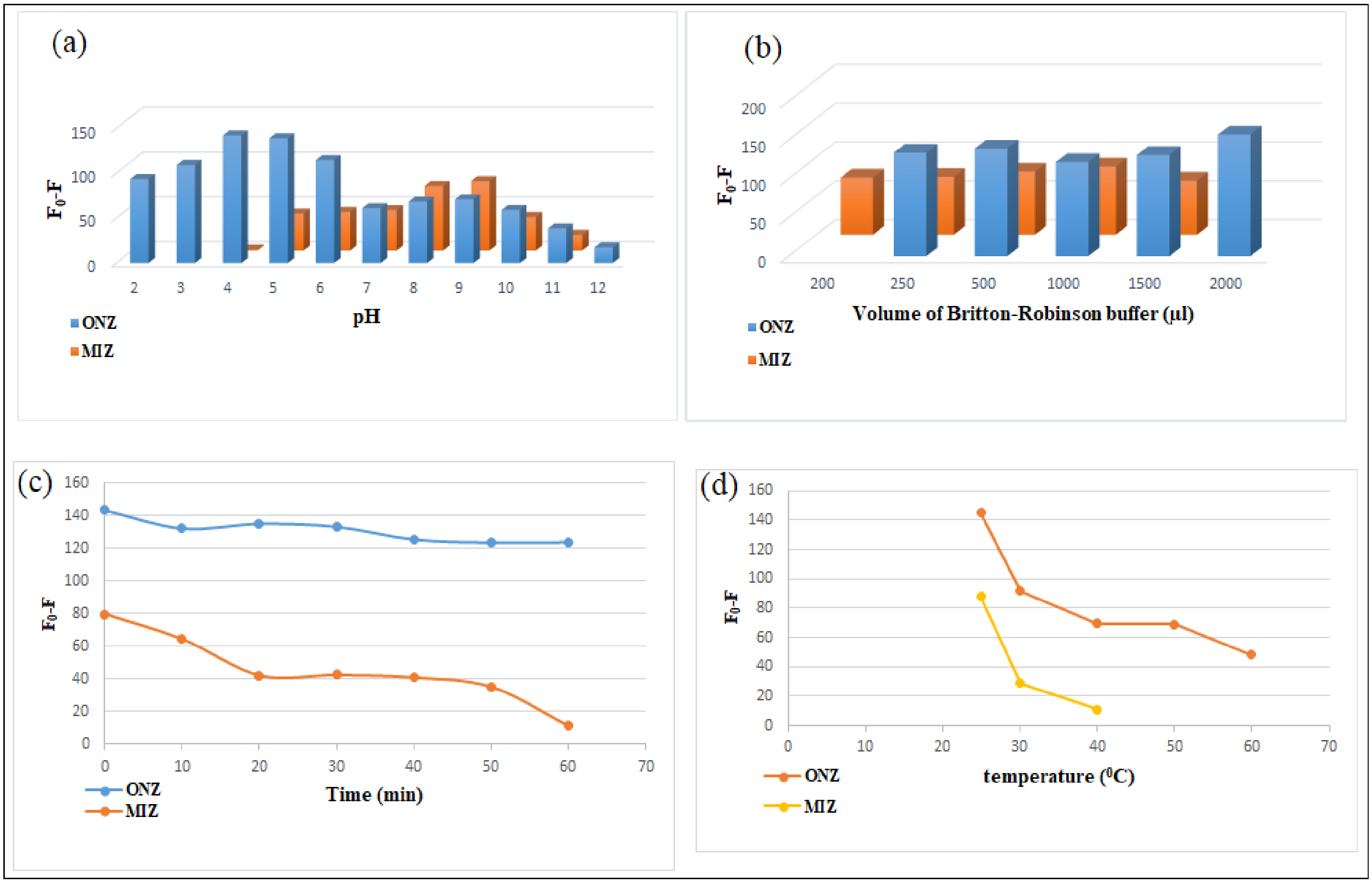
(a)
F0-F
150
100
50
0
2 3 4 5 6 7 8 9 10 11 12
pH
ONZ
MIZ
(b)
F0-F
200
150
100
50
0
200 250 500 1000 1500 2000
Volume of Britton-Robinson buffer (µl)
ONZ
MIZ
(c)
F0-F
160
140
120
100
80
60
40
20
0
0 10 20 30 40 50 60 70
Time (min)
ONZ
MIZ
(d)
F0-F
160
140
120
100
80
60
40
20
0
0 10 20 30 40 50 60 70
temperature (0C)
ONZ
MIZ

Fig. S5

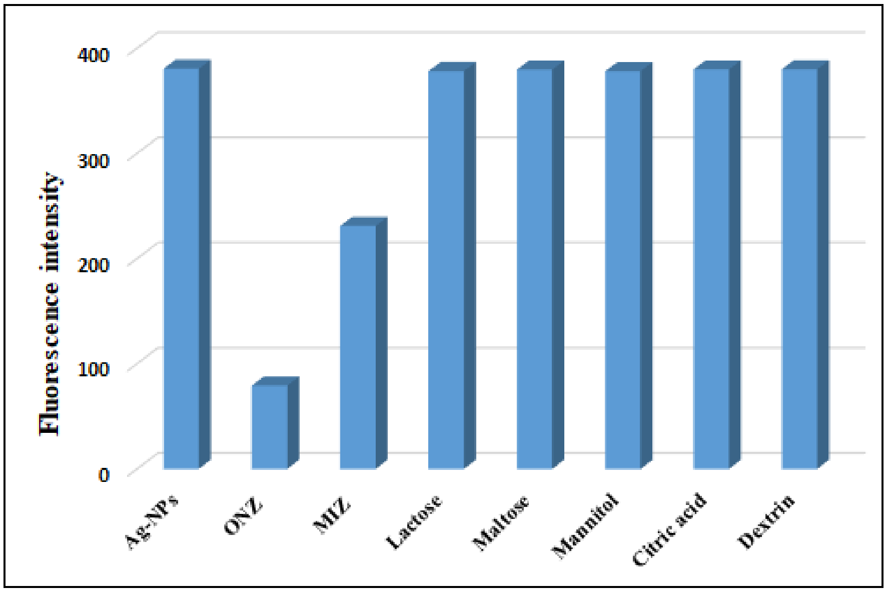
Fluorescence intensity
400
300
200
100
0
Ag-NPs
ONZ
MIZ
Lactose
Maltose
Mannitol
Citric acid
Dextrin

Fig. S6

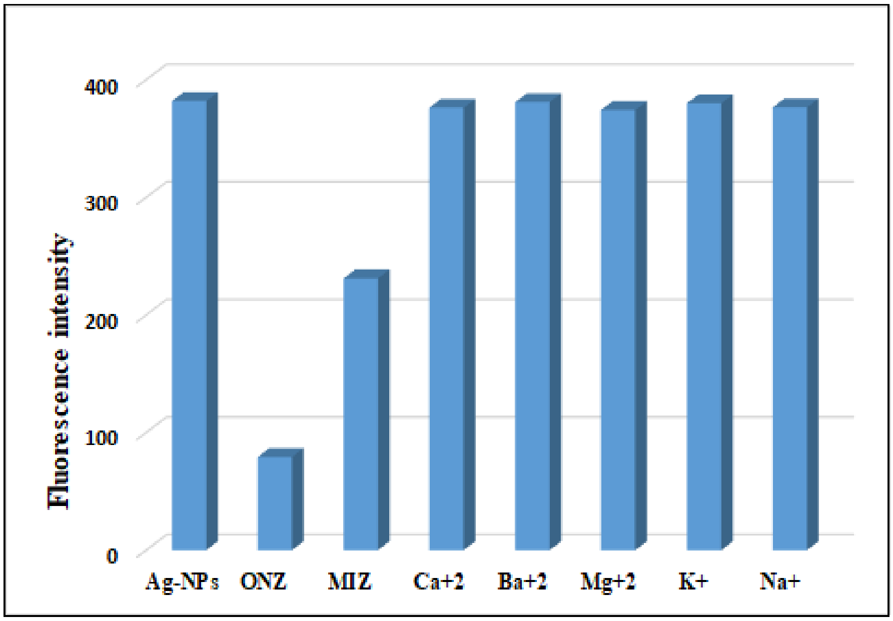
Fluorescence intensity
0
100
200
300
400
Ag-NPs
ONZ
MIZ
Ca+2
Ba+2
Mg+2
K+
Na+

Fig. S7

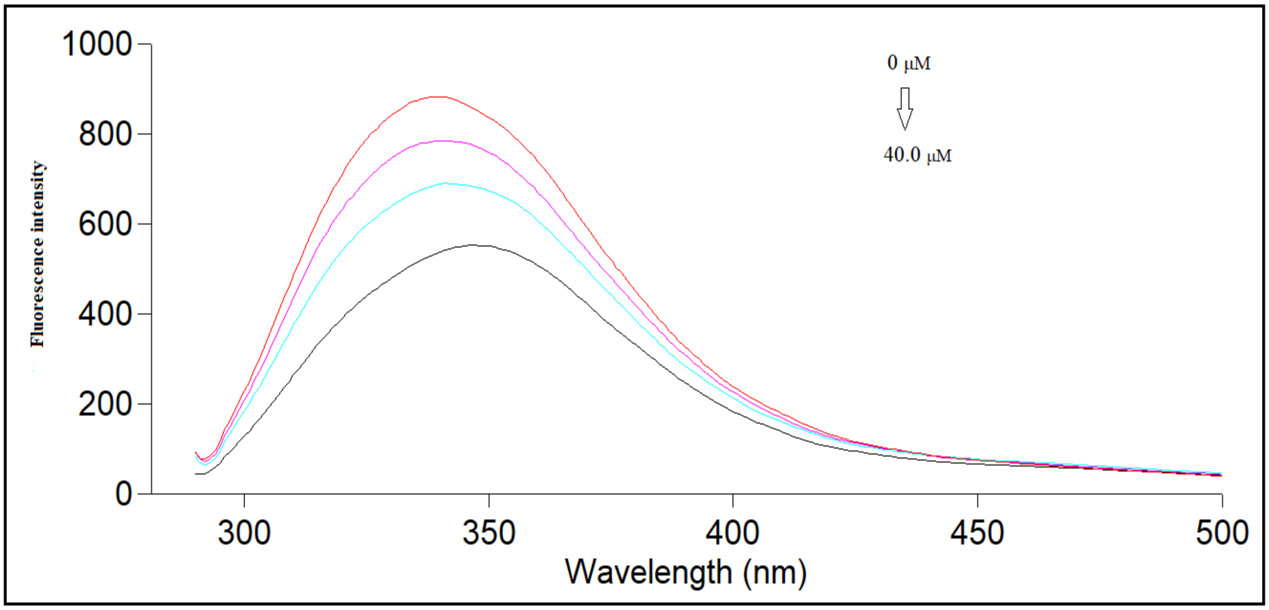
Fluorescence intensity
0
200
400
600
800
1000
300
350
400
450
500
Wavelength (nm)
0 µM
40.0 µM

Fig. S8

Table S1: Intra-day and inter-day precision data for the determination of the studied drugs by the proposed method

Analyte	Conc. taken (µM)	Intra-day precision			Inter-day precision		
		Conc. found[a] ± S.D (µM)	% RSD	% error	Conc. found[a] ± S.D (µM)	% RSD	% error
ONZ	20.0	19.79±1.03	1.04	0.60	19.85±0.54	0.55	0.32
	40.0	40.42±0.59	0.59	0.34	40.19±1.11	1.10	0.64
	60.0	59.92±0.56	0.56	0.32	60.03±0.31	0.31	0.18
MIZ	40.0	40.04±1.12	1.11	0.64	39.79±0.76	0.77	0.44
	60.0	60.36±0.35	0.34	0.20	59.79±1.11	1.11	0.64
	80.0	79.02±0.97	0.98	0.57	80.41±1.25	1.25	0.72

[a] Each result is the average of three separate determinations.

Table S2: Robustness evaluation of the proposed method

Factor	ONZ	
**1- Volume of Ag-NPs (500 µL ± 5)**	**% Recovery**	**%RSD**
**495 µL**	101.25	0.78
**500 µL**	101.74	0.79
**505 Ml**	101.42	0.49
**2- Britton-Robinson buffer pH (4 ± 0.2)**	**% Recovery**	**%RSD**
**pH = 3.8**	101.67	1.59
**pH = 4**	101.74	0.79
**pH = 4.2**	102.47	0.71
**3- Volume of Britton-Robinson buffer (2 mL ± 0.1)**	**% Recovery**	**%RSD**
**1.9 mL**	101.24	0.72
**2 mL**	101.74	0.79
**2.1 mL**	101.55	0.57
**Factor**	**MIZ**	
**1- Volume of Ag-NPs (500 µL ± 5)**	**% Recovery**	**%RSD**
**495 µL**	99.23	0.92
**500 µL**	99.02	1.92
**505 µL**	100.78	0.97
**2- Britton-Robinson buffer (pH 9 ± 0.2)**	**% Recovery**	**%RSD**
**pH = 8.8**	101.07	0.68
**pH = 9**	99.02	1.92

**pH = 9.2**	100.63	0.63
**3- Volume of Britton-Robinson buffer (1 mL ± 0.1)**	**% Recovery**	**%RSD**
**0.9 mL**	100.93	0.67
**1 mL**	99.02	1.92
**1.1 mL**	100.07	1.36
